# Peripheral Gangerene, an Unusual Presentation of Infantile Kawasaki: A Case Report and Literature Review

**DOI:** 10.1155/2021/6629405

**Published:** 2021-04-13

**Authors:** Fatemeh Tahghighi, Maryam Bakhtiari Koohsorkhi, Vahid Ziaee

**Affiliations:** ^1^Children's Medical Center, Pediatrics Center of Excellence, Tehran, Iran; ^2^Department of Pediatrics, Tehran University of Medical Sciences, Tehran, Iran; ^3^Pediatric Rheumatology Research Group, Rheumatology Research Center, Tehran University of Medical Sciences, Tehran, Iran

## Abstract

**Introduction:**

Diagnosing infantile Kawasaki disease with atypical symptoms is difficult, and it also has higher risk of coronary abnormalities which is one of the most common complications of KD. Other complications such as pericardial effusion, mitral insufficiency, congestive heart failure, myocardial systolic dysfunction, and systemic vasculitis were also reported. Peripheral gangrene and necrosis are among the rare complications of this systemic vasculitis. *Case Presentation*. We report an 8-month-old girl with prolonged fever, generalized petechial rash, cracked erythematous lips, edema, and coronary ectasia who received two doses of IVIG in another center, but short after her discharge, she started to develop a necrotic plaque on her knee. She was admitted in our hospital, and the repeat echocardiography showed sustained coronary ectasia. She received 3 doses of methylprednisolone pulse therapy and was discharged with aspirin and prednisolone. In the follow-up visits, the coronary ectasia was resolved and the necrotic ulcer was healing with a scar.

**Conclusions:**

The diagnosis of Kawasaki disease and echocardiographic evaluation of the coronary arteries should be considered in young infants with prolonged fever of unknown origin. Peripheral gangrene is a rare but important complication of infantile Kawasaki disease, although the exact mechanism in not fully understood.

## 1. Introduction

Kawasaki disease (KD) is an acute, self-limited febrile disease of unknown origin that affects children. It is now the most common cause of acquired heart disease in children in developed countries and the second most common vasculitis of childhood after Henoch–Schonlein Purpura [1]. 80% of the affected patients are younger than 4 years [2], and the peak age of incidence is between 9 and 11 months [3, 4].

Diagnosing infantile Kawasaki disease with atypical symptoms is very difficult, and it has higher risk of coronary abnormalities [5]. The algorithm recently published by the American Heart Association increases the sensitivity of KD diagnosis in infancy while maintaining specificity [6].

Coronary artery lesions are the most significant complication of KD [7], but other complications such as pericardial effusion, mitral insufficiency, congestive heart failure, myocardial systolic dysfunction, and systemic vasculitis were also reported. Peripheral gangrene and necrosis are among the rare complications of this systemic vasculitis [1, 2, 7, 8]. Early diagnosis and treatment improves the prognosis significantly and lowers the risk of coronary artery involvement and other complications [4, 6].

Here, we report a case of an 8-month-old girl with necrotic ulcer on her knee along with KD presentations.

## 2. Case Presentation

The patient is an 8-month-old girl without any significant past medical history with fever, bilateral conjunctivitis, and generalized nonpalpable petechial rash from 7 days prior to her first admission. She was treated with broad-spectrum antibiotics with the impression of meningococcemia. But, all the obtained cultures were reported negative, the fever persisted, and the patient started to develop red cracked lips and edematous extremities. The echocardiography showed coronary ectasia, and she received 2 doses of IVIG (2 gr/kg each) with the impression of Kawasaki disease, which stopped the fever, and she was discharged after 3 weeks of hospitalization in good health.

**Figure 1:**
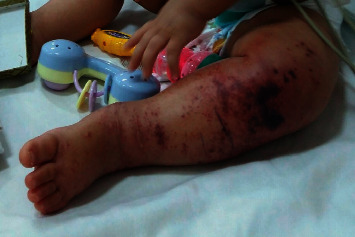
Peripheral gangrene as a presentation of Kawasaki disease.

Five days after discharge, the patient started to develop erythematous necrotic plaques on her knee (Figure 1), referred to our hospital, and got admitted for further evaluations. The lab results are as follows: WBC 13.8 × 10^3^/mm^3^ (PMN 21%), HB 9.8 g/dl, PLT 413 × 10^3^ *μ*L, CRP 3, ESR 46, ferritin 204, procalcitonin 0.2, AST 74, ALT 48, IL6 650, negative cultures, normal electrolytes. antiphospholipid (APL) antibodies, and antineutrophil cytoplasmic antibodies (ANCAs) were negative.

The chest X-ray and abdominopelvic ultrasound were normal. Doppler ultrasound showed normal flow and no sign of thrombosis, and the repeated echocardiography in our center showed sustained left coronary ectasia with a LMCA diameter of 2.9 mm and LAD diameter of 1.6 mm. The patient received 3 doses of methylprednisolone pulse therapy (30 mg/kg) and was discharged with aspirin and prednisolone.

In the 1-month follow-up of the patient, the coronary ectasia was resolved and the necrotic ulcers were healed with a scar. The 6-month and 1-year follow-up was also normal. Due to continuation of local edema in the scar location, a bone scan was performed after 6 months, which was normal.

## 3. Discussion

In the study of Alves et al. who reviewed 115 KD cases, the most prevalent complications of Kawasaki Disease were sensorineural hearing loss (33%), coronary artery aneurisms (21.7%), behavior changes and cognitive disturbances (20%), ophthalmic changes (13.2%), ataxia (9.5%), facial palsy (0.9%), peripheral gangrene and necrosis (0.9%), aortic aneurism (0.9%), and cerebral ischemia (0.9%) [9]; coronary artery lesions are the most significant complication of KD [7].

Although gangrene is a rare complication of Kawasaki disease, it carries significant morbidity for patients and requires more attention by clinicians. While we do not know the exact pathogenesis of vascular changes in KD that result in gangrene, the suggested etiologies are arteritis, arteriospasm, thrombosis of inflamed vessels, peripheral perfusion decrement [1], and, most likely, a combination of some or all of these factors.

We have found 19 similar case reports of Kawasaki patients with gangrene and a total of 24 patients in previously published Medline-indexed literatures (Table 1) (unavailable data are presented with question marks in the table content). The average age of the reported patients was 5 months, although a 4-year-old and a 22-year-old with Kawasaki and gangrene were reported as well [10, 11].

The young age of these patients could be due to delayed diagnosis of KD in this age or a possible genetic predisposition which is suggested in many studied [28]. There have been reports of ADA2 deficiency similarities and PAN in recent papers [29], and considering the close nature of KD and PAN, maybe this could branch in as well. It could explain the reported positive clinical response with anti-TNF drugs seen in patients with KD and gangrene [1, 12]. Although ADA2 deficiency is associated with endothelial damage, the exact function of this protein in endothelial integrity is still not fully understood [30].

Most of the patients were male (2 : 1), 15 patients had incomplete KD (60%), and the necrosis started from day 15–31 of the beginning of the fever. It can be assumed that since incomplete KDs are usually diagnosed later than complete KD due to the nature of their atypical symptoms and requirement for laboratory and echocardiographic evaluation for the diagnosis, the course of the disease is much more aggressive and we see more cases of peripheral necrosis in incomplete KD. The most common gangrene site was the distal part of extremities, but our patient's ulcer was on her knee. 19 patients (79%) had coronary involvement which shows the strong association between peripheral gangrene and coronary artery aneurisms. Our patient also had coronary ectasia which improved after treatment. Half of the patients had received IVIG before the 10^th^ day of the disease. Early treatment with IVIG has been shown to reduce the incidence of coronary aneurism [6, 7]; thus, it is safe to speculate that early treatment might also reduce peripheral ischemia and gangrene [13].

There has been one report of infantile KD and gangrene in a patient with factor V Leiden heterozygous mutation which could be due to the susceptibility of thrombosis formation in this patient which possibly was triggered by KD [14]; this supports the hypothesis that thrombosis plays a key role in this complication.

Different treatment approaches were suggested for these cases: methylprednisolone pulse, heparin, propranolol, warfarin, urokinase, PGE1 and prostacyclin, dipyridamole, nitroprusside, tissue plasminogen activator, topical TNG, and even caudal block [15]. Treatment with PGE1 and prostacyclin seems to have positive effects on reperfusion. However, treatment is only effective if started early in therapy [11, 13, 15–17]. von Planta et al. mentioned that their patient developed myocardial infarction after prostaglandin infusion and raise the concern that this might steal perfusion and cause ischemia in compromised coronary arteries of KD patients [18]. No proven treatment or prophylaxis approach is available in the literature. In the review of the 25 cases, only 6 experienced no significant sequel, especially amputation. It seems that once the necrosis has developed, it is really difficult to manage and control.

In our patient, considering the tissue necrosis and coronary involvement along with abnormal liver function tests, high IL6, and the previous treatment with IVIG, we gave her methylprednisolone pulse therapy which showed excellent results.

We reported a case of peripheral gangrene in an infantile Kawasaki disease to raise concern about the importance of early diagnosis, especially in atypical cases. Because this complication of KD is less mentioned, we have to report more cases to understand the epidemiology and to find the best treatment option for these patients to reduce morbidity and even mortality in such cases.

## Figures and Tables

**Table 1:** Peripheral gangrene in children with Kawasaki disease.

Reference number	Study year	Age (month)	Sex	KD criteria	KD criteria count (out of 6)	Complete/incomplete KD	Gangrene	IVIG start day	Coronary involvement	Specific treatment of gangrene	Condition of peripheral extremity
[1]	2015	4	M	Prolonged fever	1	Incomplete	Peripheral gangrene on both sides lower and right-side upper extremities	?^*∗*^	+	Methylprednisolone pulse therapy, heparin, and cyclophosphamide	Autoamputation in distal phalanxes of the right hand and left foot
	2015	6	F	Prolonged fever and desquamation	2	Incomplete	Gangrene of the middle phalanx of the right hand and finger 3	?	+	Methylprednisolone	No significant sequelae
	2015	12	M	Prolonged fever, desquamation, strawberry tongue, and LAP	4	Incomplete	Gangrene in distal phalanx of extremities	?	−	Methylprednisolone, azathioprine, heparin, and infliximab	No significant sequelae
[10]	2005	22 y/o	M	Prolonged fever, conjunctivitis, rash, LAP, and desquamation	5	Complete	Peripheral gangrene of the lower limbs	Day 55	+	Heparin, prostacyclin analogue, and prednisone	Amputation of both feet
[11]	2017	4 y/o	M	Prolonged fever	1	Incomplete	Gangrene of left side of the lower lip and left index finger	?	−	Heparin	?
[12]	2007	2	M	Prolonged fever, rash, and conjunctivitis	3	Incomplete	Left foot	Day 8	+	Heparin, topical TNG, methylprednisolone pulse, and infliximab	No significant sequelae
[13]	2006	1	M	Prolonged fever, conjunctivitis, rash, and cracked lips	4	Incomplete	Cyanosis of both the hands and feet	Day 14	+	Heparin, antithrombin III supplementation, topical TNG, methylprednisolone, dipyridamole, and abciximab	Right below-knee amputation and a left forefoot amputation and autoamputation of the left third finger tip
[14]	2018	14	M	Prolonged fever and erythematous pharynx	2	Incomplete	Right index and third finger	Day 9	+	PGE1 fresh frozen plasma methylprednisolone	Autoamputation of the second and third right fingers
[15]	1991	2	F	Prolonged fever, conjunctivitis, rash, and peripheral edema	4	Incomplete	Cyanosis of the both hands	Day 7	+	Heparin, TPA, urokinase, PGE1, and topical TNG	Autoamputation in the distal left fourth and fifth fingers
	1991	2	M	Prolonged fever and rash	2	Incomplete	Distal phalanx of the the left third finger	Day 18	+	Heparin	Autoamputation in the distal phalanx of the left third finger
	1991	3	F	Prolonged fever, conjunctivitis, LAP, cracked lips, and desquamation	5	Complete	Right foot, left hand, and left lower leg and foot	Day 13	+	Heparin, dipyridamole, and methylprednisolone	Autoamputation of the left hand and both legs
[16]	2001	3	F	Prolonged fever	1	Incomplete	Gangrene of the left forehand and left forefoot	?	+	Methylprednisolone, rTPA, and heparin	Amputation of her left hand and forefoot
[17]	1988	5	M	Prolonged fever, conjunctivitis, LAP, cracked lips, and rash	5	Complete	Both hands	−	+	Methylprednisolone, PGE1, and topical TNG	Autoamputation of the terminal phalanx of the fifth finger
[18]	1995	2	F	Prolonged fever, conjunctivitis, cracked lips, and rash	4	Incomplete	Left hand, right foot, and left foot	Day 19	+	PGE1 heparin	No significant sequelae
[19]	2011	4	M	Prolonged fever, conjunctivitis, red lips, rash, and peripheral edema	5	Complete	Fingers and toes	Day 8	+	Heparin IV, TNG, methylprednisolone, PGE1, and dipyridamole	Amputation of four distal phalanxes of the fingers
[20]	1985	4	M	Prolonged fever, rash, erythematous pharynx, palmar erythema, and desquamation	4	Incomplete	Cyanosis of the right hand, the digital tips of the left hand, and the toes of both feet	Day 21	+	Heparin ganglion block	Amputation of both hands
[21]	2012	12	M	Prolonged fever, rash, conjunctivitis, erythematous pharynx, peripheral edema, and LAP	6	Complete	Gangrene of the toes and the fingers	−	−	−	Autoamputation of the tip of the right index finger
[22]	2008	2	M	Prolonged fever, conjunctivitis, cracked lips, rash, and peripheral edema	5	Complete	Distal phalanx of the fingers and toes	Day 5	+	Methylprednisolone, MTX, heparin, and dipyridamole	No significant sequelae
[23]	2007	?	?	?		Incomplete	Both feet		+	Methylprednisolone, heparin, and prostacyclin analogue	?
[24]	1982	7	F	Prolonged fever, cracked lips, conjunctivitis, LAP, rash, and peripheral edema	6	Complete	Third and fourth fingertips of right hand	−	+	−	Autoamputation of the second and fifth fingers on the right hand
[25]	2000	3	?	Prolonged fever	1	Incomplete	+		+	Methylprednisolone heparin?	?
	2000	5	?	Prolonged fever	1	Incomplete	+		+	?	?
[26]	2019	10	F	Prolonged fever, rash, conjunctivitis, peripheral edema, and strawberry tongue	5	Complete	Toes	Day 6	−	Heparin topical TNG	?
[27]	2000	8	M	Prolonged fever, cracked lips, conjunctivitis, LAP, rash, and peripheral edema	6	Complete	Both hands and feet	Day 5	?	Heparin dipyridamole	Amputation at the tip of the right middle finger
Present case	2020	8	F	Prolonged fever, rash, cracked lips, peripheral edema, and conjunctivitis	5	Complete	Knee	Day 20	+	Methylprednisolone	No significant sequelae

?^*∗*^: not provided in the article.
